# Pure Motor Isolated Finger Palsy by Cerebral Infarction: Tips for Diagnosis by Neurological Examination

**DOI:** 10.7759/cureus.54390

**Published:** 2024-02-18

**Authors:** Yuka Nakaya, Koji Hayashi, Asuka Suzuki, Takumi Matsuyama, Mamiko Sato, Yasutaka Kobayashi

**Affiliations:** 1 Department of Rehabilitation Medicine, Fukui General Hospital, Fukui, JPN; 2 Graduate School of Health Science, Fukui Health Science University, Fukui, JPN

**Keywords:** finger palsy, precentral knob, pure motor isolated finger palsy, pseudo-peripheral neuropathy, pseudo-peripheral palsy

## Abstract

There are various causes of unilateral finger palsy. Its potential etiologies include peripheral neuropathy, carpal tunnel syndrome, and nerve root disorder due to myelopathy. In addition to them, similar paralysis has been reported in localized lesions of the cerebrum, classically referred to as pseudoperipheral palsy. In this report, we describe a case of an 80-year-old man who developed sudden clumsiness of the right fingers. Neurological examination showed muscle weakness mainly in the 1st and 2nd fingers (Medical Research Council grade 1-4) and normal reflexes in the extremities. The affected muscles were innervated by the median nerve, ulnar nerve, and radial nerve, and their nerve root levels ranged from C6 to T1. All the Phalen’s, Tinel’s, and flick signs were negative. Diffusion-weighted brain magnetic resonance imaging showed hyperintensity limited in the precentral knob on the left precentral gyrus. The etiology was diagnosed as cardiogenic embolism due to atrial fibrillation. In this report, we provide key findings for diagnosing pure motor isolated finger palsy by cerebral infarction through neurological examination.

## Introduction

There are various causes of unilateral finger palsy. Its potential etiologies include peripheral neuropathy, carpal tunnel syndrome, and nerve root disorder due to myelopathy. In addition to them, similar paralysis has been reported in localized lesions of the cerebrum, classically referred to as pseudoperipheral palsy [1,2]. The first report of pseudoperipheral palsy was described by Lhermitte in 1909 [2]. Subsequently, similar cases have been reported under various terms like pure motor monoparesis (PMM) [3], isolated hand palsy (IHP) [4], and isolated finger palsy (IFP) [5]. Some cases are accompanied by sensory disturbance or cortical symptoms among their previous reports, whereas reports of pure motor isolated finger palsy (PMIFP) caused by localized lesions in the cerebrum without other neurological symptoms except finger palsy are rare [1]. In cases of finger palsy, including PMIFP, PMM, IHP, and IFP, the cerebral localization is typically an infarct in the precentral knob (PK) region characterized by an inverted omega shape on brain images, including computed tomography (CT) or magnetic resonance imaging (MRI) [1,3-6]. While the above differential diagnosis is listed for unilateral finger palsy, there is no literature reporting detailed differential points in clinical findings, including neurological findings, because of limited reports of PMIFP by cerebral infarction. In this study, we presented a case of PMIFP localized to the first and second fingers of the right hand due to a small infarction in the PK region. In addition, we discuss key findings for the diagnosis of PMIFP related to cerebral infarction by neurological examination.

## Case presentation

An 80-year-old man with a history of diabetes and hyperlipidemia developed sudden clumsiness of the right fingers. He was a daily smoker. Vital signs monitor showed high blood pressure (178/93 mmHg) and sinus rhythm. Neurological examination showed muscle weakness mainly in the 1st and 2nd fingers (Table 1), normoreflexia in the extremities, normal sensation, and normal motor coordination. No other muscle weakness was pointed out. Phalen’s, Tinel’s, and flick signs were all negative.

**Table 1:** The result of muscle strength of the right fingers, their dominant nerves, and their nerve root levels. MRC: Medical Research Council.

Muscle	MRC grade	Dominant nerves	Nerve root
Opponens pollicis brevis muscle	3	Median nerve	C8, T1
Abductor pollicis brevis muscle	1	Median nerve	C8, T1
Adductor pollicis muscle	3	Ulnar nerve	C8, T1
Flexor pollicis longus muscle	5	Median nerve	C7, C8, T1
Flexor pollicis brevis muscle	4	Median nerve	C7, C8, T1
Abductor pollicis longus muscle	3	Radial nerve	C7, C8
Extensor pollicis brevis muscle	3	Radial nerve	C7, C8
Extensor pollicis longus muscle	3	Radial nerve	C6-8
Extensor digitorum muscle	3	Radial nerve	C6-8
Flexor digitorum superficialis muscle	5	Median nerve	C7, C8
Flexor digitorum profundus muscle	5	Median & ulnar nerves	C7, C8, T1
Extensor indicis proprius muscle	3	Radial nerve	C6-8
Flexor digiti minimi	5	Ulnar nerve	C7, C8, T1
Abductor digiti minimi	5	Ulnar nerve	C8, T1
Opponens digiti minimi	5	Ulnar nerve	C7, C8, T1
First dorsal interosseous muscle	3	Ulnar nerve	C8, T1

**Figure 1:**
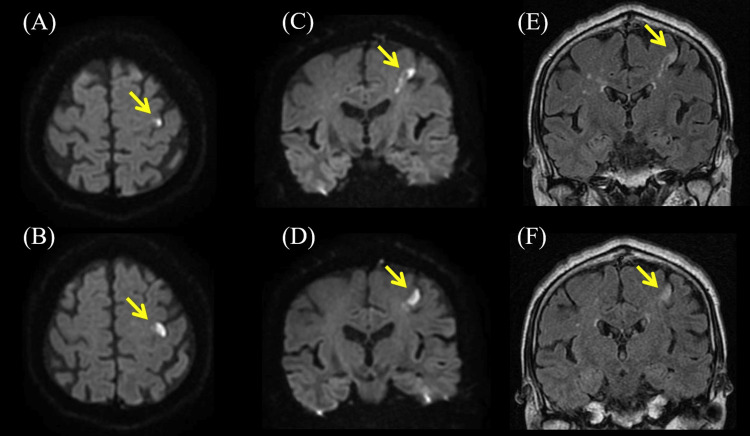
The results of brain magnetic resonance imaging (MRI). (A, B) Diffusion-weighted brain axial MRI showing hyperintensity in precentral knob (PK) on left precentral gyrus (arrowheads). (C, D) Diffusion-weighted brain coronal MRI showing hyperintensity on left precentral gyrus (arrowheads). (E, F) T2-weighted fluid-attenuated inversion recovery brain coronal MRI showing hyperintensity on the left precentral gyrus corresponded with diffusion-weighted imaging (arrowheads).

**Figure 2:**
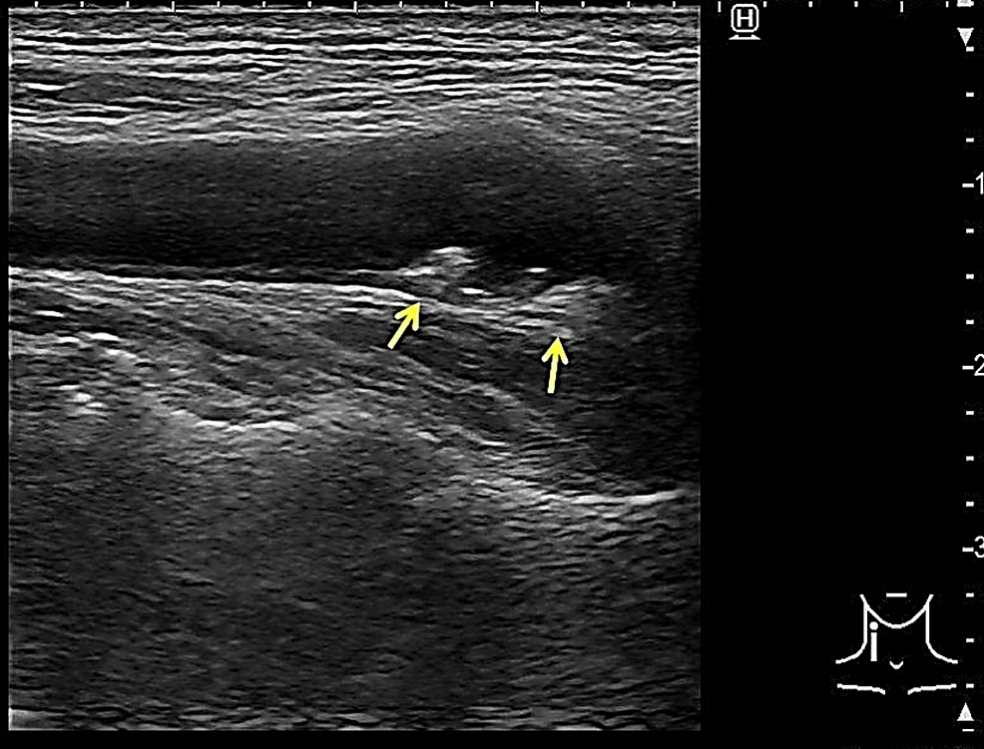
The result of carotid ultrasound. Carotid ultrasound showing plaques of the bilateral carotid bulbs (arrowheads, this echogram is the right side).

Blood tests were unremarkable except for slightly elevated C-reactive protein (0.32 mg/dL), blood glucose (118 mg/dL), and γ-glutamyl transpeptidase (75 U/L). Nerve conduction studies revealed normal results. Diffusion-weighted and T2-weighted fluid-attenuated inversion recovery brain MRI showed hyperintensity in PK on the left precentral gyrus (Figure 1). Brain magnetic resonance angiography showed no occlusion of the major artery. Transesophageal echocardiography showed no thrombus, right-to-left shunt, or aortic arch lesion. Carotid artery echocardiography revealed plaques on both bulbuses and thickening of the intima media thickness (IMT), but no increment of blood flow velocity (Figure 2).

Because the patient had a background of diabetes and hypertension, he was a current smoker, and atrial fibrillation was not detected on admission, cilostazol was prescribed and rehabilitation treatment was started on the assumption that the etiology of cerebral infarction was an artery-to-artery embolism. On day five of admission, atrial fibrillation was observed on ECG monitoring, and we diagnosed cerebral infarction in PK due to cardiogenic cerebral embolism, and the medication was changed from cilostazol to edoxaban. By day 11 of admission, the Medical Research Council (MRC) grade of the right thumb had recovered to 4 or better in all movements. On the other hand, the MRC grade of the index finger remained unchanged at 3 in extension, but otherwise recovered to 4 or better, and the patient was discharged the same day. After one month, his paralysis of the right fingers almost completely recovered.

## Discussion

We described a case of PMIFP due to cerebral infarction with paralysis limited to the first and second fingers of the right hand. The patient had no associated sensory deficits, normal reflexes, and negative Phalen's, Tinel's, and flick signs. Nerve conduction studies showed no abnormality and brain MRI revealed fresh cerebral infarction limited in PK. Atrial fibrillation was detected after hospitalization, and cardiogenic cerebral embolism was suspected as the mechanism of cerebral infarction. Anticoagulants were selected as prophylactic agents for another stroke.

Table 1 shows the muscles that were paralyzed in this case. As shown here, the innervating nerves spanned the median, ulnar, and radial nerves, and the nerve root levels ranged between the sixth cervical (C6) and first thoracic vertebrae (T1). Thus, muscle weakness of fingers related to multiple innervation or multiple cervicothoracic segmental levels cannot be explained by peripheral neuropathy. In addition, the fact that the tendon reflexes were normal cannot be explained at the level of the peripheral nerves, nerve roots, or spinal cord. Moreover, there were no findings suggestive of carpal tunnel syndrome, such as Phalen's sign, Tinel's sign, or flick signs, and if carpal tunnel syndrome was present, there would be motor and sensory deficits only in the median nerve area. Therefore, the result of neurological examination may exclude the differential diagnosis of peripheral neuropathy, radiculopathy, myelopathy, and carpal tunnel syndrome.

Of the previous reports of finger palsy in cerebral infarction in the PK region, the literature mentioning tendon reflexes is limited, but within the scope of our search, we identified reports of normal reflexes [1,7,8]. In addition, like our case, vascular disorders, including cerebral infarction, have a sudden onset, and the course of onset is also important in the differential. If patients with finger palsy present with sudden onset, normal reflexes, and finger palsy across multiple innervation and multiple nerve root levels, stroke in the PK region should be suspected.

Generally, paralysis due to stroke is accompanied by hyperreflexia in the four extremities. To understand why cerebral infarction in the PK region presents normal reflexes, it is necessary to understand the mechanism of hyperreflexia and spasticity. The pyramidal tract divides into the corticospinal tract and the corticobulbar tract [9]. Of these, the corticospinal tract sends commands to the muscles of the limbs. In the literature, the isolated involvement of the corticospinal tract does not present spasticity, but loss of dexterity, hypotonia, and hyporeflexia [10,11]. Hyperreflexia and spasticity are related to the involvement of the reticulospinal tract (RST) and vestibulospinal tract (VST) [10,11]. Alpha motor neurons, which connect to muscle spindles and receive excitatory/inhibitory inputs from RST and VST, become imbalanced in these inputs after central nervous system damage, leading to spasticity [11]. However, abnormalities in RST outflow are considered to play a major role in the genesis of spasticity in humans, while the VST, although responsible for decerebrate rigidity, appears to have a limited role [11]. In this connection, the corticoreticulospinal tract, one of the extrapyramidal motor pathways in the human brain, consists of RST and corticoreticular pathway (CRP) [12]. It is known that CRP mainly originates from the premotor cortex and ends at the pontomedullary reticular formation, leading to RST [12]. In addition, from pontine reticular formation, RST sends excitatory commands, whereas from medullar reticular formation, RST sends inhibitory commands [12]. Medullar reticular formation directly connects to CRP, but pontine reticular formation less connects to CRP [11]. Therefore, when CRP is involved, inhibitory commands to alpha motor neurons tend to be disconnected, and excitatory commands tend to be preserved [11]. Thus, excitatory commands are transmitted excessively to alpha motor neurons, resulting in spasticity and hyperreflexia. In almost all regions of the brain, the corticospinal tract and CRP are closely adjacent to each other, although in some places they run slightly separated [12]. In particular, on the surface of the brain, CRP is located in the premotor cortex anterior to the corticospinal tract [12]. Therefore, small infarctions in pure corticospinal tracts cannot affect tendon reflex, whereas cerebral infarctions in many other areas may affect not only CRP but also pyramidal tracts, causing hyperreflexia. Based on these findings, it seemed that the etiology of normal reflex in small cerebral infarctions localized to the PK region is pure involvement of the corticospinal tract, avoiding CRP leading to RST.

In addition, it is reported that the mechanism of cerebral infarction can be estimated based on the regions of the paralyzed fingers in cases with small cortical strokes [13]. According to this report, the predominant involvement of ulnar-sided fingers tends to be caused by severe proximal vessel stenosis or occlusion, whereas the predominant involvement of radial-sided fingers by emboligenic stroke [13]. In our case, the paralysis was limited to the first and second fingers, and its etiology was cardiogenic embolism, assuming the presence of atrial fibrillation. This was consistent with the result of the above report. It is interesting to note that the mechanism of infarction can be inferred from the location of the affected fingers, and this may provide useful information to clinicians.

## Conclusions

We reported a case with PMIFP by cerebral infarction. The causes of unilateral finger palsy are assumed as various diseases, including peripheral neuropathy, carpal tunnel syndrome, radiculopathy, myelopathy, and cerebrovascular disease. Key findings for the diagnosis of PMIFP related to cerebral infarction by neurological examination are finger weakness related to multiple innervation and multiple nerve root levels and normal reflex. In addition, acute onset is also important information that suggests cerebral infarction. In some cases of PMIFP, the mechanism of cerebral infarction may be estimated from the dominant area of finger paralysis.
